# Effect of Sclerosis Bands in Femoral Head Necrosis on Non‐Vascularized Fibular Grafting—A Finite Element Study

**DOI:** 10.1111/os.14199

**Published:** 2024-09-02

**Authors:** Huang Yixuan, Yuan Xinwei, Gao Feifei, Mai Jianbin, Guo Mingbin, Xi Hongzhong, Song Wei, Liu Xin, Du Bin

**Affiliations:** ^1^ Department of Orthopedics The Affiliated Hospital of Nanjing University of Chinese Medicine Nanjing China; ^2^ Department of Orthopedics Jiangsu Province Hospital of Chinese Medicine Nanjing China; ^3^ Spinal Surgery Sichuan Science City Hospital Mianyang China; ^4^ Department of Orthopedics Jiangyin Hospital of Traditional Chinese Medicine Wuxi China; ^5^ Department of Orthopedics Nanjing Jiangbei Hospital Nanjing China

**Keywords:** Finite Element Analysis, Hip Preservation, Non‐Vascularized Fibular Grafting, Femoral Head Osteonecrosis, Sclerosis Band

## Abstract

**Objective:**

Femoral head necrosis is a challenging condition in orthopaedics, and the occurrence of collapse is an important factor affecting the prognosis of femoral head necrosis. Sclerosis bands are known to influence the collapse of the femoral head, yet there is a lack of research on the biomechanical role of sclerosis bands in non‐vascularized fibular grafting surgery. This study aims to evaluate the biomechanical impact of sclerosis bands in femoral head necrosis and their role in non‐vascularized fibular grafting surgery (NVFG) using finite element analysis.

**Methods:**

We constructed 11 finite element models based on CT scan data of a normal hip joint, simulating different sclerosis band thicknesses and defect scenarios. The models were analyzed for changes in femoral head displacement and von Mises stress. We constructed a hip joint model based on CT data from a normal hip joint, and after reconstruction, assembly, and optimization using 3‐matic. We created five groups consisting of 11 finite element analysis models of the hip joint. Mesh partitioning and mechanical parameter settings were performed in ANSYS. The changes and differences in femoral head displacement and von Mises stress of these models were analyzed.

**Results:**

Increasing sclerosis band thickness led to reduced peak displacement of the femoral head by 28.6%, 42.9%, and 47.6%, and increased surface von Mises stress by 28.3%, 13.8%, and 13.0%, respectively. Post‐surgery, peak displacement decreased in all groups compared to pre‐surgery levels. Increasing sclerosis band thickness post‐surgery resulted in decreased maximum von Mises stress of the femoral head by 13.9%, 3.0%, and 8.1%. Defect volume in the defect groups correlated with increased peak displacement of the femoral head by 10.0%, 30.0%, and 100.0%, and increased surface maximum von Mises stress of the femoral head by 9.3%, 14.0%, and 15.1%.

**Conclusion:**

Sclerosis band formation exacerbates von Mises stress concentration on the femoral head surface. However, thicker sclerosis bands improve post‐NVFG stability and mechanical performance. Larger anterior lateral sclerosis band defects significantly compromise postoperative stability, increasing the risk of collapse. Protecting the anterior lateral sclerosis band during NVFG surgery is crucial.

## Graphical abstract

This study simulated the stress and displacement distribution on the surface of the femoral head after non‐vascularized fibular grafting surgery using a finite element model. The results indicated that the sclerosis band in the anterolateral region plays a mechanical supporting role for the femoral head after non‐vascularized fibular grafting surgery. This suggests that during non‐vascularized fibular grafting surgery, the sclerosis band in the anterolateral area should be avoided, as its removal may lead to the risk of early collapse postoperatively.

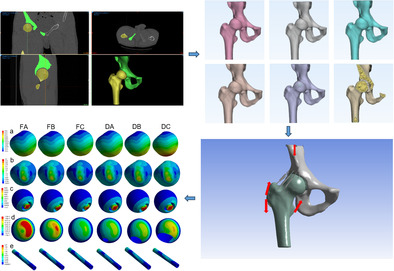

## Introduction

Osteonecrosis of the femoral head (ONFH) refers to the process of bone cell and tissue death followed by subsequent self‐repair in the femoral head due to interrupted blood flow for various reasons.^1^, ^2^ Its pathogenesis remains unclear, making ONFH a challenging condition to treat in the field of orthopaedics. It is also one of the causes of hip joint pain and functional impairment, which may progress to disability in advanced stages. Total hip replacement arthroplasty (THA) is a method for treating late‐stage ONFH. However, due to the limitations of complications associated with THA surgery and the lifespan of prostheses, patients may face multiple revisions throughout their lives, imposing significant economic burdens on themselves, their families, and society.

Therefore, early intervention in the femoral head to delay or prevent collapse is crucial, especially for young and middle‐aged patients. Non‐vascularized fibular grafting (NVFG) is an important measure for patients in the precollapse stage of ONFH. Postoperative protected weight‐bearing can alleviate symptoms, reconstruct the hip joint, and delay THA.^3^, ^4^ However, improving the efficacy of NVFG has always been a direction actively explored by clinical practitioners.

Sclerosis bands are products of self‐repair in ONFH. Their unique pathohistological formation mechanism and distinctive biomechanical characteristics make them a focal point of research in the diagnosis and treatment of ONFH.^5^ Our previous clinical research indicated that the volume ratio of the anterolateral sclerosis band is an independent protective factor against the progression of collapse after NVFG surgery. As the volume ratio of the anterolateral sclerosis band increases, the risk of collapse progression gradually decreases.^6^ Additionally, Yu et al.^7^ discovered in clinical practice that the proportion of the proximal sclerosis band in femoral head osteonecrosis is smaller in cases that have not collapsed. When the proportion of the proximal sclerosis band is less than 30%, the risk of collapse increases. However, there is currently a lack of discussion on the mechanism from a biomechanical perspective. Therefore, it is necessary to investigate the relationship between the two through finite element analysis.

The objectives of this study are: (i) to explore the effects of sclerosis bands of different thicknesses on the mechanical environment of the femoral head; (ii) to investigate the effects of sclerosis bands of different thicknesses on the mechanical environment of the femoral head post NVFG; and (iii) to explore the effects of different degrees of defects in the anterior lateral sclerosis band on the mechanical environment of the femoral head post NVFG.

## Materials and Methods

### Construction of Hip Joint Model

A male patient (36 years old, height 178 cm, weight 77 kg) with left femoral head necrosis was selected for this study. To ensure the absence of any surgery or skeletal diseases on the right side, CT data of the normal right hip joint were used to construct a three‐dimensional finite element model (FEM) of the normal hip joint. The CT scan parameters were as follows: tube voltage 120–140 kV, tube current 220–680 mA, slice thickness 1–3 mm, and reconstruction matrix 512 × 512. The scan range extended from the upper edge of the pelvis to 20 cm below the lesser trochanter. The generated image files were saved in DICOM format and copied to a personal computer using a portable hard drive. The medical software used in this study included Mimics Medical 21.0 (Materialize, Belgium), 3‐matic Medical 13.0 (Materialize, Belgium), and ANSYS Workbench 2020 (ANSYS, USA).

**FIGURE 1:**
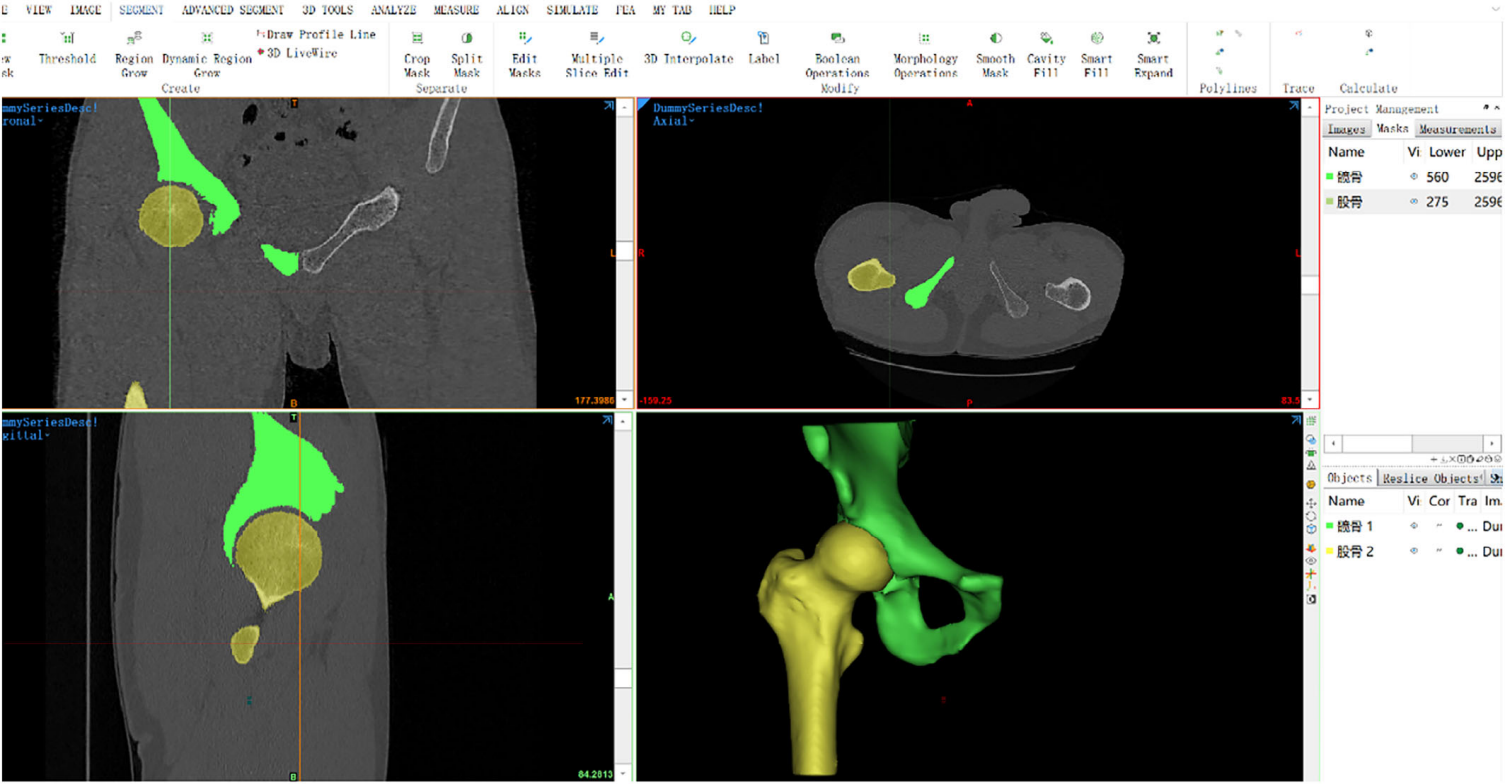
Establishment of a three‐dimensional model of the hip joint.

**FIGURE 2:**
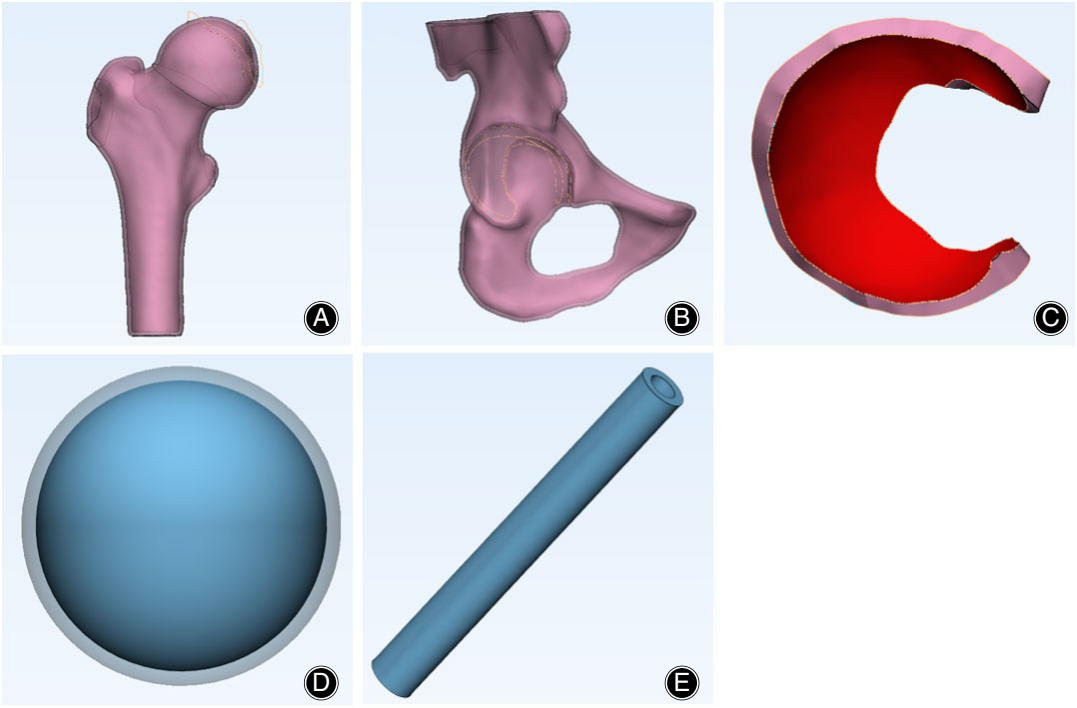
Optimization of the three‐dimensional hip joint model. (A) Femoral Head Cortical and Medullary Models; (B) Pelvic Bone Cortical and Medullary Models; (C) Cartilage Model; (D) Sclerosis Band and Necrotic Zone Models; (E) Fibular Model.

The CT images were imported into Mimics Medical 21.0, and the threshold for normal adult bone tissue was selected (226‐1666 HU) to complete the threshold segmentation (Thresholding). After region growing, the right hip bone and acetabulum were automatically separated using the CT Bone function. Layer filling (Edit mask) was performed, and the “Draw” and “Erase” tools were used to refine each layer of the images, ensuring that there were no blank areas within the hip bone and femur masks. The initial three‐dimensional model of the hip joint was obtained through three‐dimensional calculation (Calculate Part), as shown in Figure 1. The preliminary three‐dimensional model was then imported into 3‐matic Medical 13.0 software for model repair (Fix wizard) and smoothing (Local smoothing). The cortical and medullary models of the hip bone and femoral head were constructed by uniformly offsetting the selected models inward by 2 mm using the “uniform offset” command, as depicted in Figure 2A,B.

### Construction of Necrotic Zone, Sclerosis Band, Fibular Models

A spherical model with a radius of 10 mm was constructed to simulate the necrotic zone. The “Uniform offset” command was applied to create shells of sclerosis bands with three different thicknesses around the necrotic area by offsetting the sphere outward by 0.1 mm, 0.3 mm, and 0.5 mm, respectively (the groups were named Sclerosis Band Group A, B, and C, respectively). The thickness of the sclerosis band was determined based on the volume ratio of sclerosis band observed in previous clinical studies.^6^ Sclerosis band defects were created by using Boolean operations to remove regions with radii of 2.5 mm, 5.0 mm, and 7.5 mm from the anterolateral surface of the 0.5 mm sclerosis band (the groups were named Sclerosis Band Defect Group A, B, and C, respectively). The necrotic zone and sclerosis band are shown in Figure 2D. A hollow cylindrical model of the fibular with a radius of 5.0 mm, height of 85 mm, and wall thickness of 2.0 mm was simulated based on dimensions provided by the manufacturer, based on the varying thicknesses of the sclerosis band, the groups can be named Fibular Implantation Group A, B, and C (Figure 2E). The cartilage was created by using the “Create curve” command to trace the edge of the acetabular cartilage, followed by the “Extrude” command to obtain a preliminary cartilage model. Finally, the cartilage was refined between the acetabulum and the femoral head using the “Boolean subtraction” command with Boolean operations to optimize the cartilage model (Figure 2C).

### Hip Joint Model Positioning

According to the definition of JIC C1 classification,^8^ the necrotic zone and sclerosis band were moved to the anterior lateral region of the femoral head. In the anteroposterior view, the necrotic zone extended laterally to the edge of the acetabulum but did not exceed it. The position of the fibular was determined according to the procedure of fibular support surgery,^4^ with one end of the fibular located 1.5 cm below the greater trochanter and the other end positioned at the center of the necrotic zone.

### Mesh Partitioning of the Hip Joint Model

**FIGURE 3:**
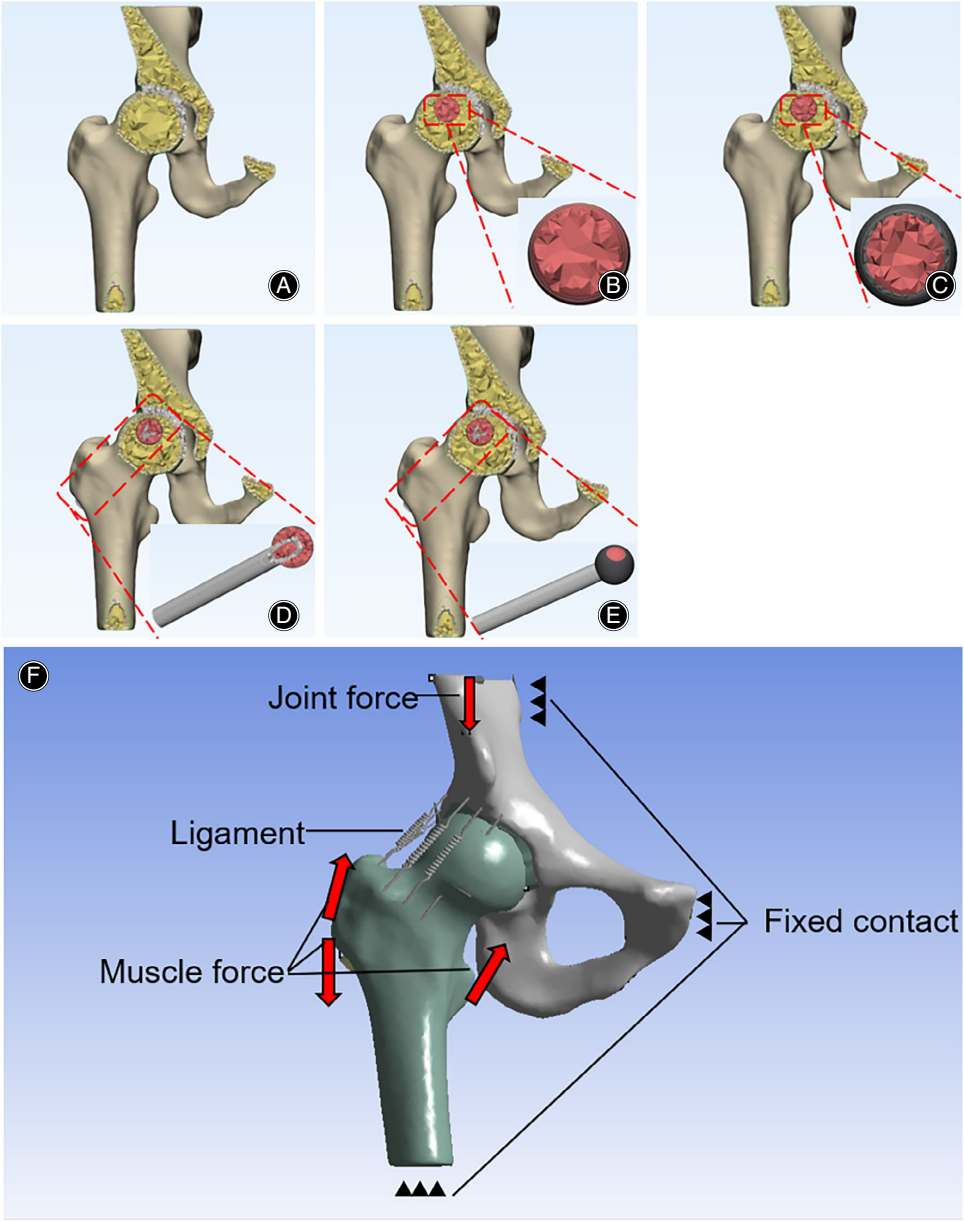
Hip joint mesh model and its constraints and mechanical loads. (A) Normal Hip Joint Mesh; (B) Femoral Head Necrosis Mesh; (C) Sclerosis Band Formation Mesh; (D) Fibular Implantation Mesh; (E) Sclerosis Band Defect Mesh; (F) Model constraints and mechanical loads.

All structural models within the hip joint were selected, and the “Merge” command was utilized to combine all hip joint models into a single collection. Subsequently, the “Create Self‐intersection” command was employed to intersect the individual models, ensuring that shared surfaces were preserved while any redundant surfaces were eliminated. The “Adaptive Remesh” command was used to optimize the surface mesh after which the “Create Volume Remesh” command was used to create the volume mesh (tetrahedral elements). These steps were repeated to build models for the normal hip joint, femoral head necrosis model, sclerosis band model, fibular implantation model, and sclerosis band defect model. The specific groupings are detailed in Table 1, and the models for each group are illustrated in Figure 3A–E.

**TABLE 1:** Finite element model grouping settings.

Group	Abbreviation	Necrotic zone radius (mm)	Sclerosis band thickness (mm)	Defect radius (mm)
Normal hip joint	Normal	‐	‐	‐
Femoral head necrosis group	Necrosis	10	‐	‐
Sclerosis band group A	SA	10	0.1	‐
Sclerosis band group B	SB	10	0.3	‐
Sclerosis band group C	SC	10	0.5	‐
Fibular implantation group A	FA	10	0.1	‐
Fibular implantation group B	FB	10	0.3	‐
Fibular implantation group C	FC	10	0.5	‐
Sclerosis band defect group A	DA	10	0.5	2.5
Sclerosis band defect group B	DB	10	0.5	5.0
Sclerosis band defect group C	DC	10	0.5	7.5

### Material Assignment

The models constructed in 3‐matic were imported into ANSYS Workbench 2020 for static structural analysis. The material properties of cortical bone, trabecular bone, cartilage, necrotic bone, and sclerosis band were obtained from the literature: E_cortical_ = 15100 MPa, E_trabecular_ = 445 MPa, E_cartilage_ = 10.5 MPa, E_lesion_ = 125 MPa, E_sclerosis band_ = 5500 MPa; ν_cortical_ = 0.3, ν_trabecula_r = 0.22, ν_cartilage_ = 0.45, ν_lesion_ = 0.152, ν_sclerosis band_ = 0.3.^9^, ^10^ Table 2 summarizes the material properties of these models.

**TABLE 2:** Material assignment.

Property	Elastic modulus (MPa)	Poisson's ratio
Ilium cortical	15,100	0.3
Ilium trabecular	445	0.22
Femur cortical	15,100	0.3
Femur trabecular	445	0.45
Cartilage	10.5	0.45
Sclerosis band	5500	0.3
Necrotic bone	124.6	0.15
Fibular	15,100	0.3

### Load Application and Boundary Conditions

For the boundary conditions, as described by Wen et al.,^11^ nodes at the pubic symphysis, the upper edge of the pelvic bone, the sacroiliac joint, and the distal femur were fully fixed to prevent any translation or rotation. Following the settings of the hip joint interface by Xiong et al.,^12^ the joint surface between the cartilage and the femoral head was defined as frictionless, and the interface between the necrotic bone and normal bone or normal bone tissue was defined as fully bonded. Referring to Shi et al.,^13^ the coefficient of friction for the tantalum rod against the surrounding bone was set at 0.88; however, considering that the fibula does not have the threaded structure of a tantalum rod, this study adjusted the coefficient of friction down to 0.6. In accordance with Bunn et al.,^14^ the surrounding ligaments of the hip joint, including the iliofemoral, pubofemoral, and ischiofemoral ligaments, were simulated using spring models, with three spring models representing each ligament. The stiffness of the spring models simulating different ligaments is detailed in Table 3. Joint pressure and muscle forces, including the gluteus medius, tensor fasciae latae, and iliopsoas muscles, were applied to the hip joint. Based on the research by Komistek et al.,^15^ a force equivalent to 240% of body weight was loaded onto the hip joint surface in the direction of gravity, with a magnitude of 1800N. The muscle force loading settings were referenced from the study by Chen et al.,^16^ where the gluteus medius and tensor fasciae latae act on the lateral aspect of the greater trochanter, and the iliopsoas muscle acts on the lesser trochanter, defined as component forces in the XYZ directions, with magnitudes of 292N, 300N, and 188N, respectively, as shown in Table 4. The final model setup is illustrated in Figure 3F.

**TABLE 3:** Spring longitudinal stiffness table.

Simulated ligaments	Stiffness (N/mm)
Iliacofemoral ligament	3560
Iliofemoral ligament	1706
Ischiofemoral ligament	719

**TABLE 4:** Joint forces and muscle forces settings.

Load	Size (N)	X‐direction (N)	Y‐direction (N)	Z‐direction (N)
Joint force	1800	0	0	−1800
Abductor muscle force	300	102	0	281
Iliopsoas muscle force	188	−128.2	−19.1	136.2
Gluteus medius muscle force	292	0	0	−292

### Validation of Three‐Dimensional Finite Element Model

**FIGURE 4:**
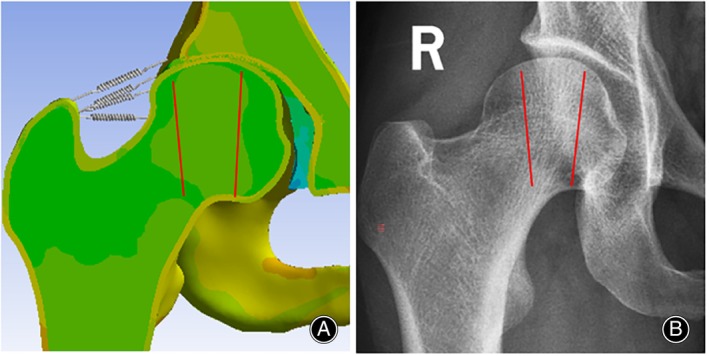
Finite element model validation. (A) Proximal Femoral Stress Transmission Pathway; (B) Orientation of Pressure Osteons in the Proximal Femur.

The mechanical performance of the model was evaluated by comparing it with existing research results. This study found that the region of von Mises stress in the model is located in the anterior lateral region of the femoral head, with a maximum von Mises stress (1.96 MPa) similar to that reported by Li et al.^17^ (1.92 MPa). Additionally, the main principal stresses within the femoral head estimated by the finite element model (Figure 4) exhibit shapes and regions consistent with X‐ray images.

### Statistical Analysis

All data were statistically analyzed using SPSS (version 25.0, SPSS Inc., Chicago, IL, USA) and were analyzed with descriptive statistical methods. We conducted descriptive statistical measurements for key variables of the femoral head cortical surface, sclerosis band, necrotic area, and fibular strut, including maximum von Mises stress and maximum displacement deformation. By comparing these key variables and visually representing them through charts and graphs, the aim was to provide intuitive data visualization.

## Results

### Impact of Sclerosis Band on the Internal Structure of the Femoral Head

**FIGURE 5:**
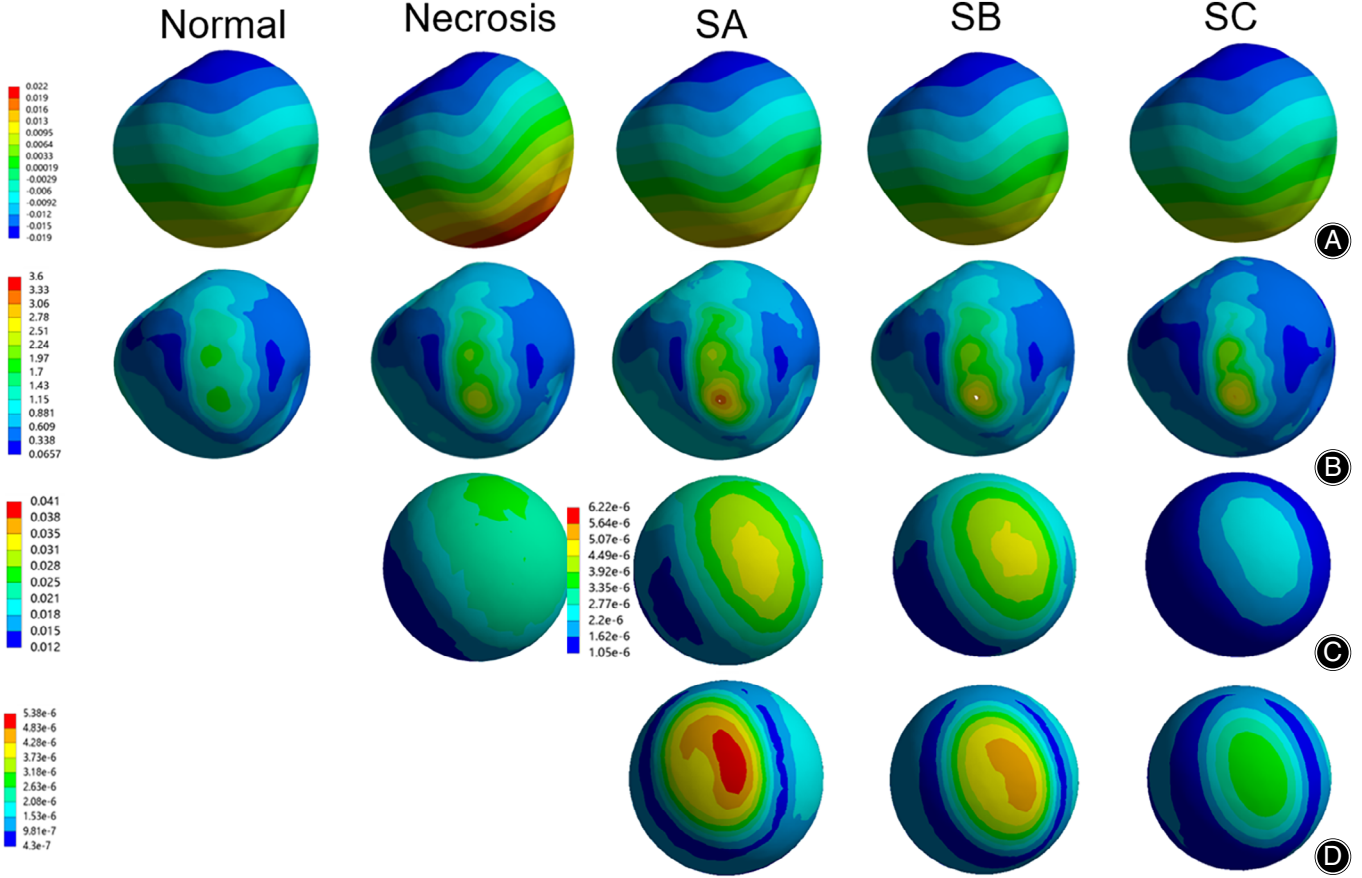
Preoperative directional displacement cloud and von Mises stress distribution plots. (A) Femoral head surface directional displacement; (B) Femoral head surface von Mises stress; (C) Necrotic area von Mises stress; (D) Sclerosis band von Mises stress. SA. Sclerosis band Group A; SB. Sclerosis band Group B; SC. Sclerosis band Group C.

The peak displacement of the femoral head was 1.2 × 10^−2^ mm in the normal group and 2.1 × 10^−2^ mm in the necrosis group. The sclerosis band group showed a decreasing trend in the peak displacement compared to the necrosis group, with values of 1.5 × 10^−2^ mm, 1.2 × 10^−2^ mm, and 1.1 × 10^−2^ mm, respectively, representing reductions of 28.6%, 42.9%, and 47.6% compared to the necrosis group (Figures 5A, 7A and Table 5). The maximum von Mises stress of the femoral head was distributed in the anterior lateral region in all groups, with the maximum von Mises stress on the femoral head surface being 1.96 MPa in the normal group and 2.47 MPa in the necrosis group. The surface von Mises stresses in sclerosis band groups A, B, and C were 3.17 MPa, 2.81 MPa, and 2.79 MPa, respectively, representing increases of 28.3%, 13.8%, and 13.0% compared to the necrosis group (Figures 5B, 7B and Table 6).

**TABLE 5:** Peak directional displacement for each group.

Categories	Peak displacement (mm)				
	Preoperative group				
	Normal	Necrosis	SA	SB	SC
Femoral head	1.2 × 10^−2^	2.1 × 10^−2^	1.5 × 10^−2^	1.2 × 10^−2^	1.1 × 10^−2^

	Postoperative group					
	FA	FB	FC	DA	DB	DC
Femoral head	1.1 × 10^−2^	1.0 × 10^−2^	1.0 × 10^−2^	1.1 × 10^−2^	1.3 × 10^−2^	2.0 × 10^−2^

Abbreviations: DA, sclerosis band defect group A; DB, sclerosis band defect group B; DC, sclerosis band defect group C; FA, fibular implantation group A; FB, fibular implantation group B; FC, fibular implantation group C; SA, sclerosis band group A; SB, sclerosis band group B; SC, sclerosis band group C.

**TABLE 6:** Peak von Mises stress values for each group.

Categories	Peak von Mises stress (MPa)				
	Preoperative group				
	Normal	Necrosis	SA	SB	SC
Femoral head	1.96	2.47	3.17	2.81	2.79
Necrosis area		4.13 × 10^−2^	6.76 × 10^−6^	6.2 × 10^−6^	4.76 × 10^−6^
Sclerosis band			5.38 × 10^−6^	4.97 × 10^−6^	3.15 × 10^−6^

	Postoperative group					
	FA	FB	FC	DA	DB	DC
Femoral head	2.73	2.73	2.58	2.82	2.94	2.97
Necrosis area	3.30 × 10^−3^	3.20 × 10^−3^	3.00 × 10^−3^	3.22 × 10^−3^	3.51 × 10^−3^	3.66 × 10^−3^
Sclerosis band	1.59 × 10^−5^	1.21 × 10^−5^	7.74 × 10^−6^	8.70 × 10^−6^	9.08 × 10^−6^	1.22 × 10^−5^
Fibula	2.40	2.59	2.81	2.47	2.30	2.19

Abbreviations: DA, sclerosis band defect group A; DB, sclerosis band defect group B; DC, sclerosis band defect group C; FA, fibular implantation group A; FB, fibular implantation group B; FC, fibular implantation group C; SA, sclerosis band group A; SB, sclerosis band group B; SC, sclerosis band group C.

The maximum von Mises stress in the necrotic area for both the necrosis and sclerosis band groups was located at the center of the necrotic area. The von Mises stress magnitude in the necrotic area for the necrosis group was 4.13 × 10^−2^ MPa, while for Sclerosis Band Groups A, B, and C, it was 6.76 × 10^−6^ MPa, 6.22 × 10^−6^ MPa, and 4.76 × 10^−6^ MPa, respectively, showing a significant decrease compared to the necrosis group (Figures 5C, 7C and Table 6). The maximum von Mises stress on the sclerosis band in the sclerosis band for groups A, B, and C were 5.38 × 10^−6^ MPa, 4.97 × 10^−6^ MPa, and 3.15 × 10^−6^ MPa, respectively, showing a decreasing trend (Figures 5D, 7D and Table 6).

### Stress Impact on Femoral Head Structure after Fibular Implantation

**FIGURE 6:**
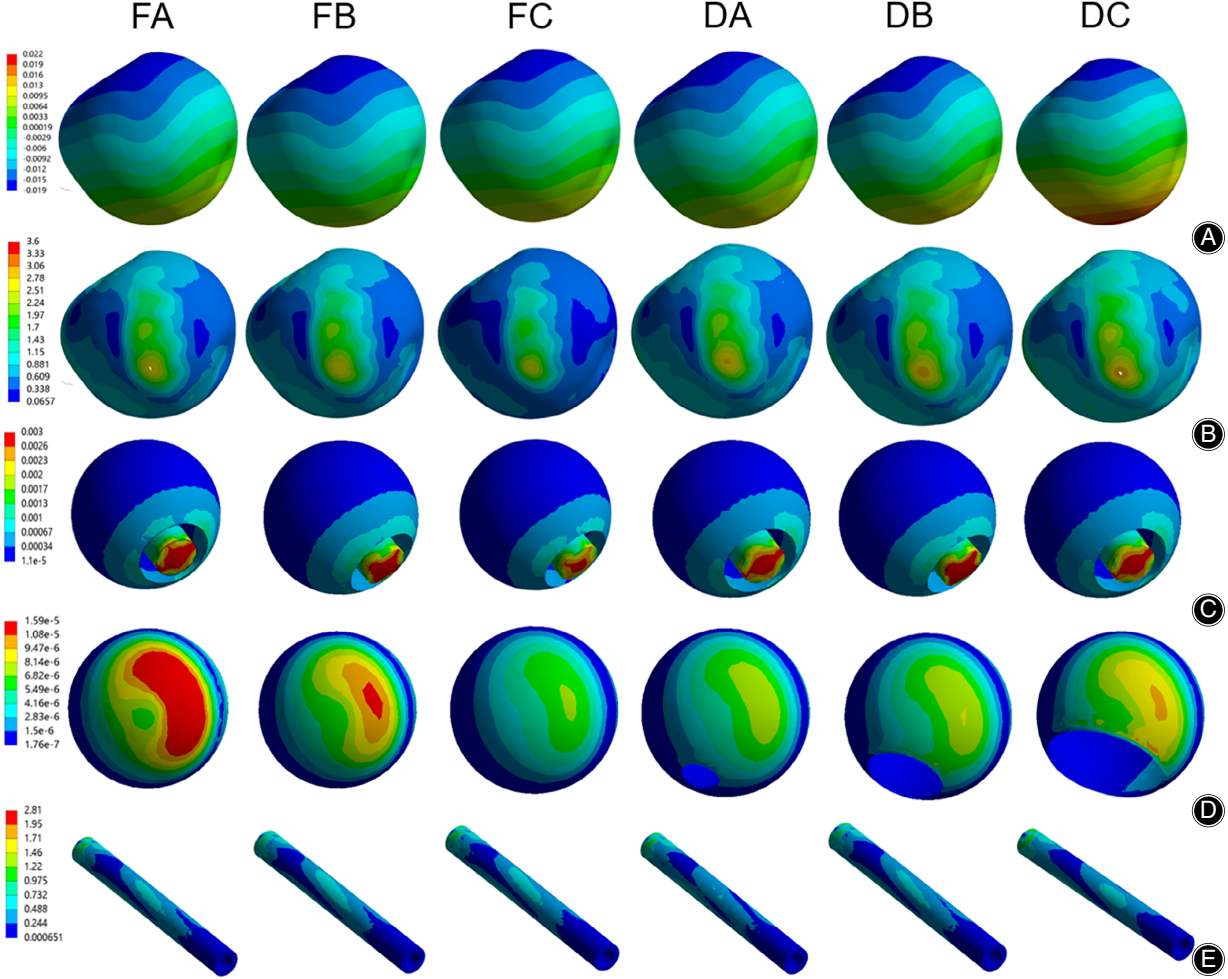
Postoperative directional displacement cloud and von Mises stress distribution plots. (A) Femoral Head Surface Displacement; (B) Femoral Head Surface Mises Stress; (C) Necrotic Area Mises Stress; (D) Sclerosis Band Mises Stress; (E) Fibular Mises Stress. FA. Fibular Implantation Group A; FB. Fibular Implantation Group B; FC. Fibular Implantation Group C; DA. Sclerosis Band Defect Group A; DB. Sclerosis Band Defect Group B; DC. Sclerosis Band Defect Group C.

**FIGURE 7:**
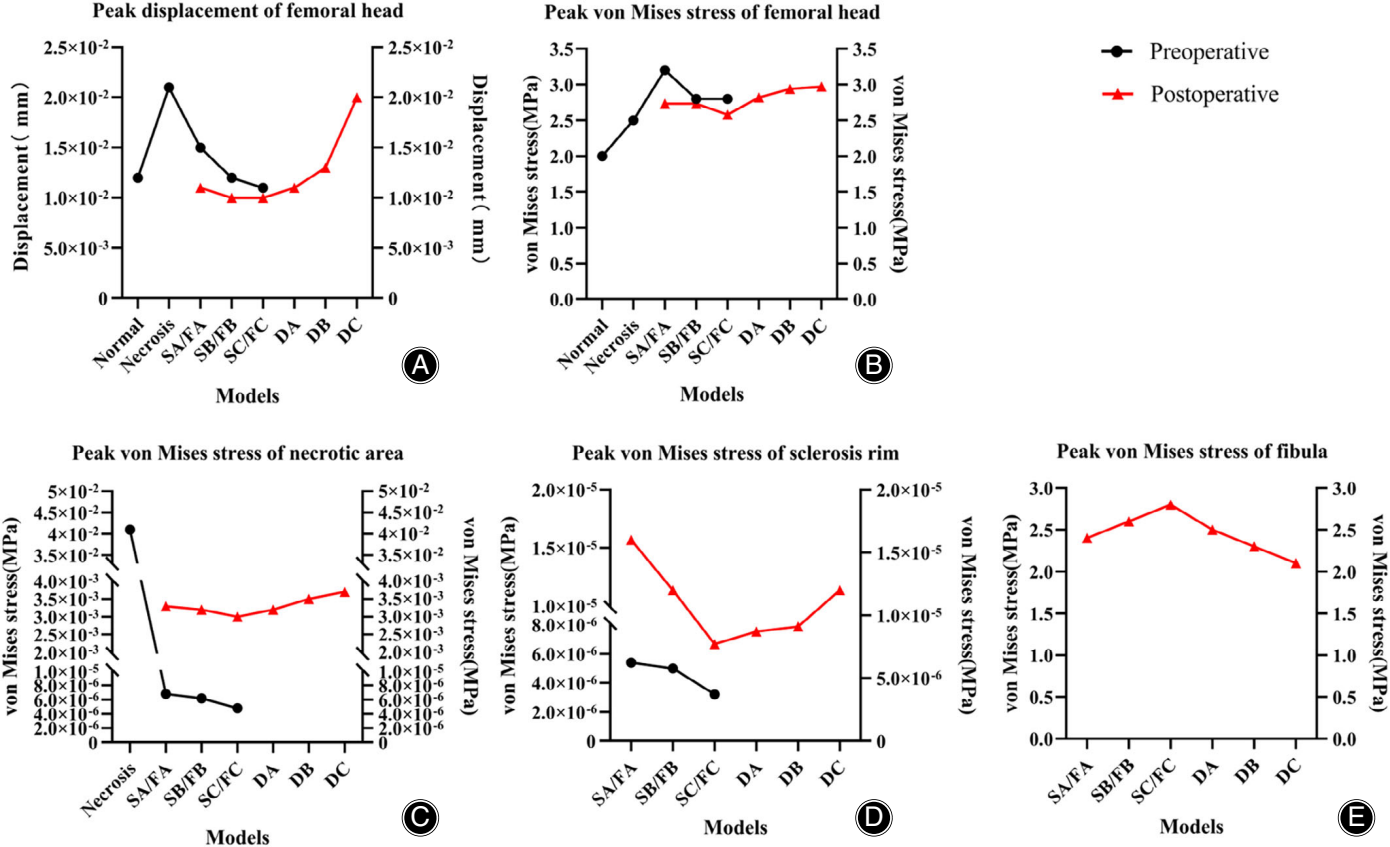
Preoperative and Postoperative Peak Directional Displacement and Peak von Mises stress dual line charts. (A) Preoperative and Postoperative Femoral Head Surface Peak Displacement; (B) Preoperative and Postoperative Maximum Mises Stress on Femoral Head Surface; (C) Preoperative and Postoperative Maximum Mises Stress in Necrotic Area; (D) Preoperative and Postoperative Maximum Mises Stress in Sclerosis Band; (E) Postoperative Maximum Mises Stress on Fibular. SA. Sclerosis band Group A; SB. Sclerosis band Group B; SC. Sclerosis band Group C; FA. Fibular Implantation Group A; FB. Fibular Implantation Group B; FC. Fibular Implantation Group C; DA. Sclerosis Band Defect Group A; DB. Sclerosis Band Defect Group B; DC. Sclerosis Band Defect Group C.

The displacement of Fibular Implantation Groups A, B, and C were 1.1 × 10^−2^ mm, 1.0 × 10^−2^ mm, and 1.0 × 10^−2^ mm, which was lower than the necrosis group's 2.1 × 10^−2^ mm, but still higher than the normal group's 2.1 × 10^−2^ mm level (Figures 6A, 7A and Table 5). The maximum von Mises stress in the cortical bone of the femoral head in Fibular Implantation Groups A, B, and C appeared on the anterior lateral side, with values of 2.73 MPa, 2.73 MPa, and 2.58 MPa, respectively. This represented a decrease of 13.9%, 3.0%, and 8.1% compared to Sclerosis Band Groups A, B, and C, but the postoperative von Mises stress remained higher than the necrosis group and did not return to the level of the normal group (Figures 6B, 7B and Table 6).

The maximum von Mises stress in the necrotic area of the femoral head in Fibular Implantation Groups A, B, and C appeared at the center of the necrotic area and the fibular implantation site, with maximum von Mises stress values of 3.30 × 10^−3^ MPa, 3.20 × 10^−3^ MPa, and 3.00 × 10^−3^ MPa, respectively. This represented an increase of 529.5 times, 472.4 times, and 629.3 times compared to Necrosis Groups A, B, and C, respectively (Figures 6C, 7C and Table 6). The corresponding maximum von Mises stress values of the sclerosis band in Fibular Implantation Groups A, B, and C were 1.59 × 10^−5^ MPa, 1.21 × 10^−5^ MPa, and 7.74 × 10^−6^ MPa, respectively. This represented an increase of 2.0 times, 1.4 times, and 1.5 times compared to Necrosis Groups A, B, and C, respectively (Figures 6D, 7C and Table 6). The maximum Mises stress of the fibular was 2.40 MPa, 2.59 MPa, and 2.81 MPa for Groups A, B, and C, respectively (Figures 6E, 7E and Table 6).

### Impact of Sclerosis Band Defects on Femoral Head Structure

The peak displacement on the cortical surface of the femoral head in Defect Groups A, B, and C was 1.1 × 10^−2^ mm, 1.3 × 10^−2^ mm, and 2.0 × 10^−2^ mm, respectively, compared to Fibular Implantation Group C, representing increases of 10.0%, 30.0%, and 100.0%, respectively (Figures 6A, 7A and Table 6). The maximum von Mises stress on the cortical surface of the femoral head was concentrated on the anterior lateral part of the cortical bone, with maximum von Mises stress values of 2.82 MPa, 2.94 MPa, and 2.97 MPa for Defect Groups A, B, and C, respectively. This represented increases of 9.3%, 14.0%, and 15.1%, respectively, compared to Implantation Group C (Figures 6B, 7B and Table 6).

The maximum Mises stress in the necrotic area of the femoral head in Defect Groups A, B, and C was 3.22 × 10^−3^ MPa, 3.51 × 10^−3^ MPa, and 3.66 × 10^−3^ MPa, respectively, representing increases of 7.3%, 17.0%, and 22.0%, respectively, compared to Implantation Group C (Figures 6C, 7C and Table 6). The corresponding maximum von Mises stress values of the Sclerosis Band Defect Groups A, B, and C were 8.70 × 10^−6^ MPa, 9.08 × 10^−6^ MPa, and 1.22 × 10^−5^ MPa, respectively, representing increases of 12.4%, 17.3%, and 57.6%, respectively, compared to Fibular Implantation Group C (Figures 6D, 7D and Table 6). The corresponding maximum von Mises stress values of the fibular in Defect Groups A, B, and C were 2.47 MPa, 2.30 MPa, and 2.19 MPa, respectively, representing decreases of 12.1%, 18.5%, and 22.0%, respectively, compared to Fibular Implantation Group C (Figures 6E, 7E and Table 6).

## Discussion

Our study has yielded several notable findings. Firstly, we observed that the formation of the sclerotic band in ONFH reduces surface displacement of the femoral head but exacerbates stress concentration on its surface. With increasing thickness of the sclerotic band, the surface stress on the femoral head gradually decreases. Secondly, post‐fibular implantation leads to some improvement in the mechanical condition of the femoral head. Lastly, when there is a defect in the anterior lateral sclerotic band, the mechanical support and surface stress on the femoral head deteriorate further post‐fibular implantation, and the degree of deterioration is positively correlated with the defect area. Based on our study, we propose that defects in the lateral sclerotic band may be one of the reasons for the failure of NVFG surgery, thus corroborating the findings of our previous clinical research from a biomechanical perspective. Therefore, it is essential to provide targeted protection to the anterior lateral sclerotic band during surgery in order to enhance the clinical efficacy of hip‐preserving surgery.

### Relationship between Sclerotic Band and Femoral Head Collapse

The formation of the sclerosis band is the result of a combination of pathophysiological and biomechanical mechanisms. In terms of biomechanics, the formation of the sclerosis band is a product that follows Wolff's Law. Based on Wolff's theory of bone remodeling, the distribution of trabecular bone develops along the direction of bone stress, and changes in stress can cause the growth, modeling, and repair of trabecular bone.^18^ Chen et al.^16^ found that the sclerosis band forms around the necrotic area, proving that the formation of the sclerosis band is an adaptive response in the process of bone remodeling. In previous studies, the role of sclerosis bands in the collapse of osteonecrosis of the femoral head has been a subject of debate. Yang et al.,^19^ through examination of 28 femoral head necrosis specimens, found that the primarily generated necrotic area adjacent to the necrotic‐viable interface is the main site of femoral head necrosis fractures. Brown et al.^20^ suggested that stress is typically concentrated within the infarct, near the necrotic‐repair interface surrounding the necrotic area. Motomura et al.^21^ observed that collapse is often accompanied by fractures, which occur at the junction between thickened reparative trabeculae and necrotic trabeculae. These studies collectively indicate that the necrotic‐repair region may be associated with collapse. However, biomechanical research by Yu^9^ and Chen^16^ both support the notion that the presence of a sclerosis band can reduce stress on the surface of the femoral head. Clinical observations by Yu et al.,^7^ who measured the proximal sclerosis band edge ratio in 101 patients with femoral head necrosis, found that when the proximal sclerosis band ratio is >30%, the risk of collapse is lower, and when it is <30%, the risk of collapse is higher.

This study addresses the controversy in this field from two aspects. On one hand, the appearance of the sclerosis band further exacerbates the stress concentration on the surface of the femoral head necrosis. However, with the increase in the thickness of the sclerosis band, the surface stress of the femoral head shows a decreasing trend. The surface stress of the femoral head in Sclerosis Band Groups A, B, and C were 3.17 MPa, 2.81 MPa, and 2.79 MPa, respectively, which were increases of 28.3%, 13.8%, and 13.0% compared to the pure necrosis group. On the other hand, as the volume of the sclerosis band increases, there is also a downward trend in the maximum stress value of the femoral head, indicating that the internal stability of the femoral head is to some extent restored. Based on the phenomena observed in this study and the research of others, we believe that the difference in stiffness between the sclerosis band and the necrotic area causes the sclerosis band to bear a larger proportion of the load. In addition, bone adapts and reshapes itself in response to the mechanical forces it experiences. The formation and remodeling of the sclerosis band may represent an adaptive response to increased stress, aimed at restoring structural integrity and more evenly distributing stress. Therefore, changes in stiffness and stress adaptation are potential biomechanical mechanisms at play. However, it should be noted that whether the sclerosis band can play a role in preventing collapse also depends on the morphology of the sclerosis band and the location and size of the necrotic area.

### Mechanical Mechanisms of Fibular Grafting in the Treatment of Femoral Head Osteonecrosis

Current finite element analysis suggests that the biomechanical principle of fibular strut grafting is to reduce stress concentration on the anterolateral aspect and to restore the biomechanical transfer pathway from the top of the femoral head to the femoral neck.^10^, ^20^, ^22^, ^23^ Zhou et al.^10^ simulated joint loading in six cases of femoral head necrosis through finite element modeling, and the results indicated that the stress on the anterolateral aspect was restored postoperatively, with a reduction of 22.41%–23.35% compared to the preoperative state. The biomechanical transfer pathway from the top of the femoral head to the junction of the head and neck was reconstructed, resulting in a lower risk of cortical bone collapse postoperatively. The fibula bore the main load, and the stress on the cortical bone was reduced. The results of this study show that postoperatively, the stress on the surface of the femoral head was reduced by 3.0%–13.9% compared to the preoperative sclerosis band group, which is slightly lower than the extent reported in other literature.^10^, ^22^ However, the directional displacement of the femoral head surface was close to that of normal femoral heads, indicating a significant restoration of stability within the femoral head. This result also confirms that one of the biomechanical effects of fibular grafting is to reduce the stress concentration on the anterolateral aspect of the femoral head in necrosis.

However, to date, no researchers have focused on the role of the sclerosis band in this surgical procedure. This study further found that the maximum stress in the necrotic area and the sclerosis band postoperatively was significantly higher than preoperatively, especially the maximum stress in the necrotic area, which increased by 529.78 times, 472.35 times, and 628.76 times compared to Sclerosis Band Groups A, B, and C preoperatively, respectively. This indicates that the stress shielding effect of the sclerosis band on the necrotic area was significantly weakened after fibular grafting, and the stress from the surface of the femoral head was transmitted to the necrotic area. Moreover, the sclerosis band not only bears the stress on the surface of the femoral head in the early postoperative period but also assists in the long‐term postoperative reconstruction of the stress transfer pathway by helping to transfer stress through the fibular strut.

### The Guiding Role of Different Sclerosis Band Defects on Clinical Strategy

Our previous clinical research found that the volume ratio of the anterolateral column sclerosis band is a protective factor against early collapse after fibular implantation surgery. The lower the volume of the anterolateral column sclerosis band as a proportion of the total volume of the anterolateral column, the higher the probability of early collapse after surgery.^6^ To validate this clinical finding from a biomechanical perspective, this study designed three models with varying degrees of defects in the anterolateral area sclerosis band. The results showed that as the volume of the defect increased, the equivalent stress on the cortical surface of the femur exhibited an increasing trend, with values of 2.82 MPa, 2.94 MPa, and 2.97 MPa, representing increases of 9.3%, 14.0%, and 15.1%, respectively, compared to the fibular implantation group C. The peak displacements were 0.011 mm, 0.013 mm, and 0.020 mm, respectively, which correspond to increases of 10.0%, 30.0%, and 100.0% compared to the fibular implantation group C. These results indicate that defects in the anterolateral sclerosis band area weaken the mechanical repair effect of fibular implantation surgery on the femoral head and increase the risk of femoral head collapse.

Therefore, the clinical significance of this study lies in two aspects. On one hand, by integrating the results of previous clinical studies, the volume of the sclerosis band can be assessed to predict the likelihood of successful fibular implantation surgery, helping to select the optimal treatment method for patients. For instance, patients with a smaller volume of the sclerosis band might be considered for hip preservation surgeries such as “trapdoor” procedures that thoroughly remove dead bone to provide adequate support. On the other hand, in specific clinical surgical operations, surgeons should avoid excessive debridement of the anterolateral region to prevent defects in the anterolateral sclerosis band, thereby providing early stable support to the anterolateral femoral head.

### Limitations and Future Perspectives

One of the limitations of this study is the simplification of bone structure and materials. The bone materials in this study were assumed to be linearly elastic and homogeneous. Additionally, the cortical and trabecular bones of the hip joint were also assumed to be homogeneous, which may alter the natural behavior of bones. Another limitation is the idealized design of the femoral head necrosis model and fibular grafting model, as the actual morphology of necrotic areas, sclerotic bands, and fibular within the femoral head is more complex in clinical practice. Finally, this study only performed mechanical performance analysis on a single type of model without conducting large‐sample repeated experiments. The conclusions drawn are descriptive and not the result of statistical analysis. To obtain more accurate biomechanical analysis results, the development of more precise finite element models and their statistical validation should be the direction for future research.

## Conclusion

In summary, our study indicates that the anterolateral sclerosis band plays a role in enhancing mechanical support for the femoral head in fibular grafting surgery, with the effect being stronger as the thickness of the sclerosis band increases. Furthermore, when defects occur in the anterolateral sclerosis band, the mechanical support is weakened. Therefore, in clinical practice, the thickness of the anterolateral sclerosis band could be observed to assist in predicting the likelihood of collapse after fibular grafting surgery. At the same time, it is recommended that surgeons avoid excessive debridement of the anterolateral region of the femoral head during fibular grafting surgery to prevent defects in the anterolateral sclerosis band.

## Conflict of Interest Statement

The authors declare no competing interests.

## Ethical Statement

This retrospective study was approved by the Institutional Review Board of The Affiliated Hospital of Nanjing University of Chinese Medicine hospital (2023NL‐001‐01) before collecting data.

## Author Contributions

All authors had full access to the data in the study and take responsibility for the integrity of the data and the accuracy of the data analysis. Study concept and design: D.B., L.X, and H.Y.X. Acquisition of data: Y.X.W, G.G.F., M.J.B, and G.M.B. Analysis and interpretation of the data: X.H.Z. Drafting of the manuscript: S.W. Critical revision of the manuscript for important intellectual content: D.B. and L.X. Obtained funding: D.B and L.X Administrative, technical, and material support: G.F.F. Study supervision: D.B.

All authors listed meet the authorship criteria according to the latest guidelines of the International Committee of Medical Journal Editors, and all authors are in agreement with the manuscript.
